# Increasing cell culture density during a developmental window prevents fated rod precursors derailment toward hybrid rod-glia cells

**DOI:** 10.1038/s41598-023-32571-y

**Published:** 2023-04-13

**Authors:** Ivana Barravecchia, Chiara De Cesari, Viviana Guadagni, Giovanni Signore, Edoardo Bertolini, Serena Gea Giannelli, Francesca Scebba, Davide Martini, Mario Enrico Pè, Vania Broccoli, Massimiliano Andreazzoli, Debora Angeloni, Gian Carlo Demontis

**Affiliations:** 1 Department of Pharmacy, University of Pisa, Via Bonanno Pisano, 6, 56126 Pisa, Italy; 2 Scuola Superiore Sant’Anna, Pisa, Italy; 3 Department of Biology, University of Pisa, Pisa, Italy; 4 Fondazione Pisana per la Scienza, San Giuliano Terme, Italy; 5 Donald Danforth Plant Science Center, St. Louis, USA; 6 San Raffaele Hospital, Milan, Italy; 7 Institute of Neuroscience, National Research Council of Italy, Milan, Italy

**Keywords:** Neuronal development, Ion channels in the nervous system, Gliogenesis, Transcriptomics

## Abstract

In proliferating multipotent retinal progenitors, transcription factors dynamics set the fate of postmitotic daughter cells, but postmitotic cell fate plasticity driven by extrinsic factors remains controversial. Transcriptome analysis reveals the concurrent expression by postmitotic rod precursors of genes critical for the Müller glia cell fate, which are rarely generated from terminally-dividing progenitors as a pair with rod precursors. By combining gene expression and functional characterisation in single cultured rod precursors, we identified a time-restricted window where increasing cell culture density switches off the expression of genes critical for Müller glial cells. Intriguingly, rod precursors in low cell culture density maintain the expression of genes of rod and glial cell fate and develop a mixed rod/Muller glial cells electrophysiological fingerprint, revealing rods derailment toward a hybrid rod-glial phenotype. The notion of cell culture density as an extrinsic factor critical for preventing rod-fated cells diversion toward a hybrid cell state may explain the occurrence of hybrid rod/MG cells in the adult retina and provide a strategy to improve engraftment yield in regenerative approaches to retinal degenerative disease by stabilising the fate of grafted rod precursors.

## Introduction

The vertebrate retina extracts the most relevant features from the visual scene for further processing by higher visual structures^1^. Retina operation relies on three-dimensional neural networks, neatly organised in layers populated by neurons with sharply different morphologies and functional properties^2,3^. In the retina, neurons and glial cells stem from a common progenitor^4,5^, whose developmental potential progressively restricts to generate stage-specific intrinsically different progenitors, which give birth to a limited repertoire of retinal cell types (recently reviewed in^6–8^).

Transcription factors (TFs) in progenitor cells provide the intrinsic cues setting the fate of their postmitotic daughter cells, and additional specification steps may play a role in stabilising the fate of immature postmitotic precursors. Indeed, extrinsic factors such as cell culture density may promote a fate reassignment, although limited to the cell types generated by a given precursor at a given developmental time^9–11^. For instance, a reduced number of rod precursors may increase amacrine or bipolar cells, but rod precursors may not convert to early-born cone precursors.

At variance with this notion, late-born rod precursors lacking the postmitotic specification signals provided by TFs such as Neural Retina Leucine Zipper (*Nrl*)^12,13^ or Nuclear Receptor Subfamily 2 Group E Member 3 (*Nr2e3*)^14,15^ may undergo a fate diversion toward early-born blue cones^16^. It is presently unclear whether the lack of Nrl or Nr2e3 in postmitotic rod precursors leads to their true reassignment to the blue cone fate or a fate derailment toward a hybrid rod-cone cell type^17^ with reduced viability^18–22^. It is important to note that intrinsic signals provided by TFs, such as Nrl, may also depend on extrinsic factors provided by chemical cues, such as retinoic acid, which may promote rod formation by inducing Nrl transcription^23^.

The transcriptomic profile of rod precursors isolated at postnatal day 4 (PN4)^24^ reveals their somewhat hybrid properties, with the concurrent expression of genes common to their parent progenitors and those critical for the development of Müller glial (MG) cells. These data may indicate that postmitotic rod precursors retain the potential for diversion toward a hybrid rod-glial cell fate despite rod precursors and MG cells hardly coexisting in the progeny of terminally-dividing progenitors positive for the basic helix-loop-helix (bHLH) transcription factor Olig^25^.

To investigate whether extrinsic factors may affect the fate of postmitotic rod photoreceptor precursors, we monitored the impact of cell culture density on the expression of selected genes by single-cell qRT-PCR in rod precursors identified using both molecular and functional criteria.

We report that cells isolated from postnatal day 0 (PN0) retinas of NRL-GFP^+^ mice and cultured for up to 8 days in vitro (DIV) (PN0/DIV8) at low cell density keep expressing genes critical for MG cells development (*c-Kit* and *Mcam*) and acquire some electrophysiological features of MG cells. Moreover, PN0/DIV8 rod precursors expressing MG genes also express the rod-specific gene rhodopsin (*Rho*) and the hyperpolarisation-activated cyclic nucleotide-gated gene (*Hcn1*), which codes for a cationic channel common to all primary sensory neurons^26^. Interestingly, an increase in the cell culture density selectively suppresses *c-Kit* and *Mcam* expression by PN0/DIV4 rod precursors without affecting the expression of photoreceptor markers.

The notion of intrinsic and extrinsic factors interplaying in preventing the derangement toward a hybrid cell state may impact translational research for regenerative approaches to inherited retinal degenerations.

## Results

### Rod precursors express genes pertaining to other cell fates

A transcriptional shift at postnatal day 6 (PN6) precedes outer segment (OS) generation in rods^27^ and cones^28^. Therefore, we analyzed transcriptome profiles in sorted GFP^+^ rod precursors cells isolated from retinas of Nrl-GFP^+^ transgenic mice, which selectively express GFP in rod precursors^29^, over a time window better centred on PN6 than currently available data^24^, between postnatal days 4 (PN4) and 8 (PN8), i.e., when most rod precursors had been generated (PN4), or outer segments (OS) start appearing (PN8).

**Figure 1:**
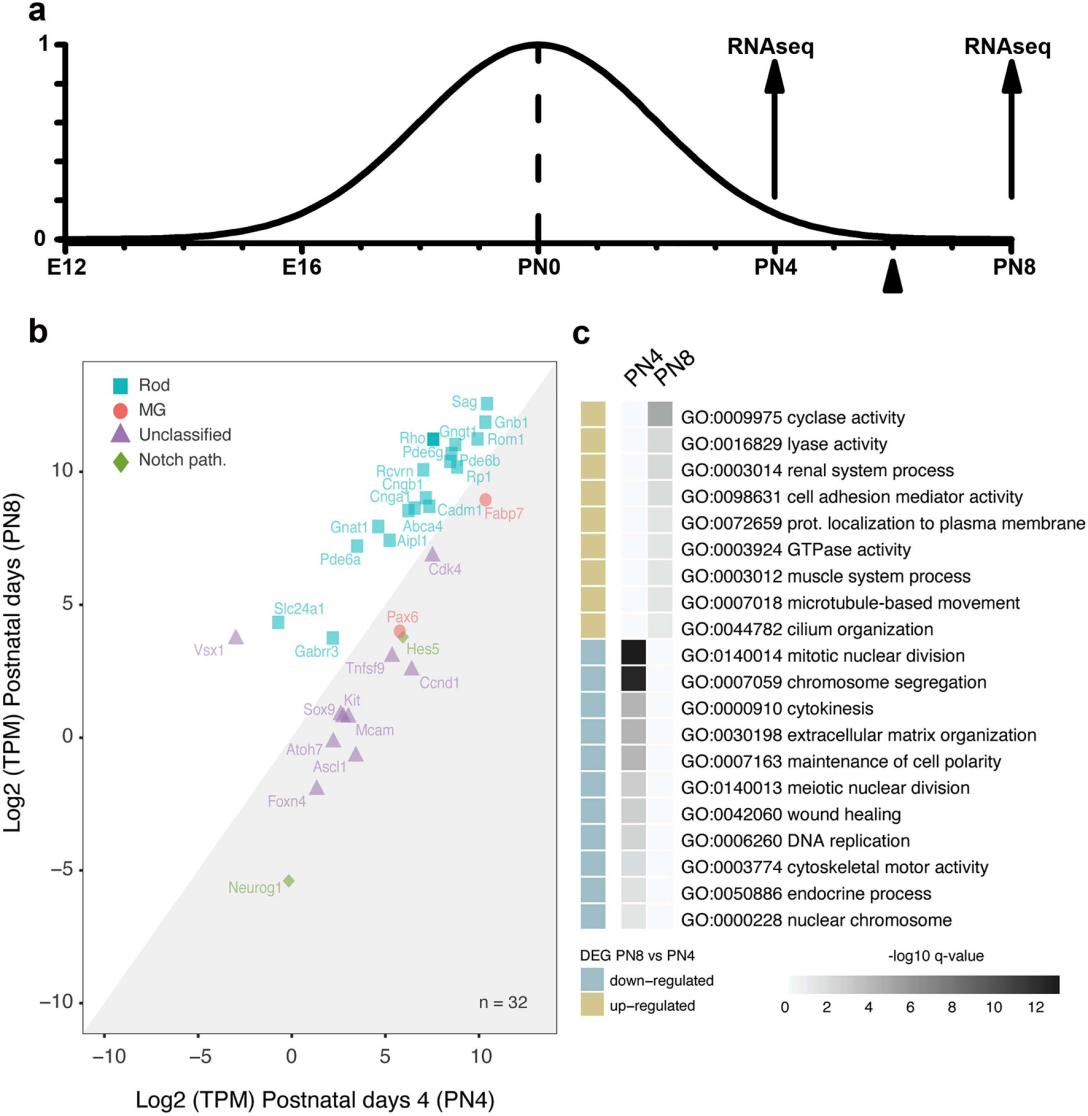
Transcriptome comparison of PN4 and PN8 Nrl:GFP^+^ rod precursors. (**a**) Schematic drawing of the time course of mouse rod precursors generation. The Y-axis plots the rate of rod precursor generation in arbitrary units. Precursors appear around embryonic day 13 (E13), the rate of generation peaks around the day of birth (PN0), and the process ends around postnatal day 6 (PN6). The arrowhead indicates the time of transcriptional shift in rod precursors. Arrows at PN4 and PN8 indicate the time of GFP^+^ rod precursors sorting for the evaluation of transcriptional changes by RNAseq analysis. (**b**) Scatter plot of log_2_ Transcripts per Million reads (TPM) at PN8 vs Log_2_ (TPM) at PN4. Each gene is plotted with X and Y coordinates representing normalized expression at PN4 and PN8, respectively. (**c**) Heatmap representing statistically significant GO over-represented classes associated with differentially expressed genes (fold change > |1.5| and false discovery rate = 0.05).

The analysis revealed 3762 differentially expressed genes (DEGs), using as a criterion a log2 fold change > |1.5| (Supplementary Table S1). Table S1 inspection reveals the upregulation of genes expressed in adult photoreceptors (*Rho*, *Gnat1*, *Gngt1*, *Pde6a*, *Pde6b*, *Pde6g*, *Aipl1*, *Cnga1*, *Slc24a1*, *Abca4*, *Sag*, *Rcvrn*) and cone bipolar cells (*Vsx1*), along with those coding for the metabotropic glutamate receptor and the ion channels expressed by on-type bipolar cells (*Grm6* and *Trpm1*). Down-regulated genes include those involved in proliferation and progenitor development and those downregulated during Muller glia (MG) development^30,31^. Figure 1b plots up/down-regulated genes for rod precursors (cyan squares) that proceed from PN4 to PN8 (i.e. PN8 vs PN4), indicating an increased expression, particularly in rod-specific and photoreceptor-specific genes (selected according to the dataset_S07 in^32^). Conversely, downregulation of genes expressed in adult MG (*Pax6* and *Fabp7*, red circles) and in the Notch pathway that plays a critical role in MG development^33^ (*Neurog* and *Hes5*, green diamonds) was also observed. Purple triangles in Fig. 1b plot genes not expressed in either adult rods or MG (unclassified) and involved in earlier^34^ and late^35^ phases of retinogenesis (such as *Pax6* and *Kit*, respectively), Muller glial cell maturation from retinal progenitors^31^ (*Cdk4* and *Ccnd1*), neurogenic factors (*Atoh7*, *Foxn4*, *Ascl1*), Notch downstream effector *Sox9*^31^, or Notch-independent adult MG (*Mcam*)^36^.

The heatmap in Fig. 1c summarizes Gene Ontology (GO) analysis of terms most significantly changed during the PN4 to PN8 transition. Among the most relevant upregulated terms, we found Cilium organization, Cyclase activity, and GTPase activity, whereas the most represented downregulated terms included Mitotic nuclear division, Chromosome segregation, Extracellular matrix organization, Establishment or maintenance of cell polarity (see Supplementary Table S2). Overall, independently of sorting criteria, photoreceptor and rod-specific genes represented the categories most relevantly upregulated in the time window analysed. Indeed, an over-representation test with Benjamini–Hochberg correction indicates 12 genes coding for rod-specific phototransduction components in Supplementary Table S3 (*Rho*, *Gnat1*, *Gngt1*, *Pde6a*, *Pde6b*, *Pde6g*, *Aipl1*, *Cnga1*, *Slc24a1*, *Abca4*, *Sag*, *Rcvrn*) are overrepresented among those upregulated at PN8 (P = 5.89 × 10–5, see Transcriptome analysis in “Materials and methods”).

These observations suggest rod precursors maturation does not proceed straightforwardly to adult rods until PN4, in agreement with the notion that fate stabilization occurs after PN4. However, it is presently unknown whether this transcriptional pattern may provide a base for rod precursors developmental plasticity in response to environmental cues.

In Fig. 1b, among the unclassified DEGs coding for transmembrane proteins, which may sense environmental cues affecting rod precursors maturation, we noted the stem cell factor receptor KIT Proto-Oncogene, Receptor Tyrosine Kinase (*c-Kit* or CD117), the Melanoma cell adhesion molecule (*Mcam* or CD146), and the Tumor necrosis factor ligand superfamily member 9 (*Tnfsf9* or CD137L). In addition, besides *Notch1*^33^, *Mcam* expression is critical for MG cell development^36^, and *c-Kit* is a marker for late progenitor cells generating rod and MG cells^35^. These observations suggest that GFP^+^ rod precursors retain a transcriptional profile with elements common to the MG fate for several days from the initial fate assignment, indicating a slow transition of postmitotic rod precursors to adult rods.

**Figure 2:**
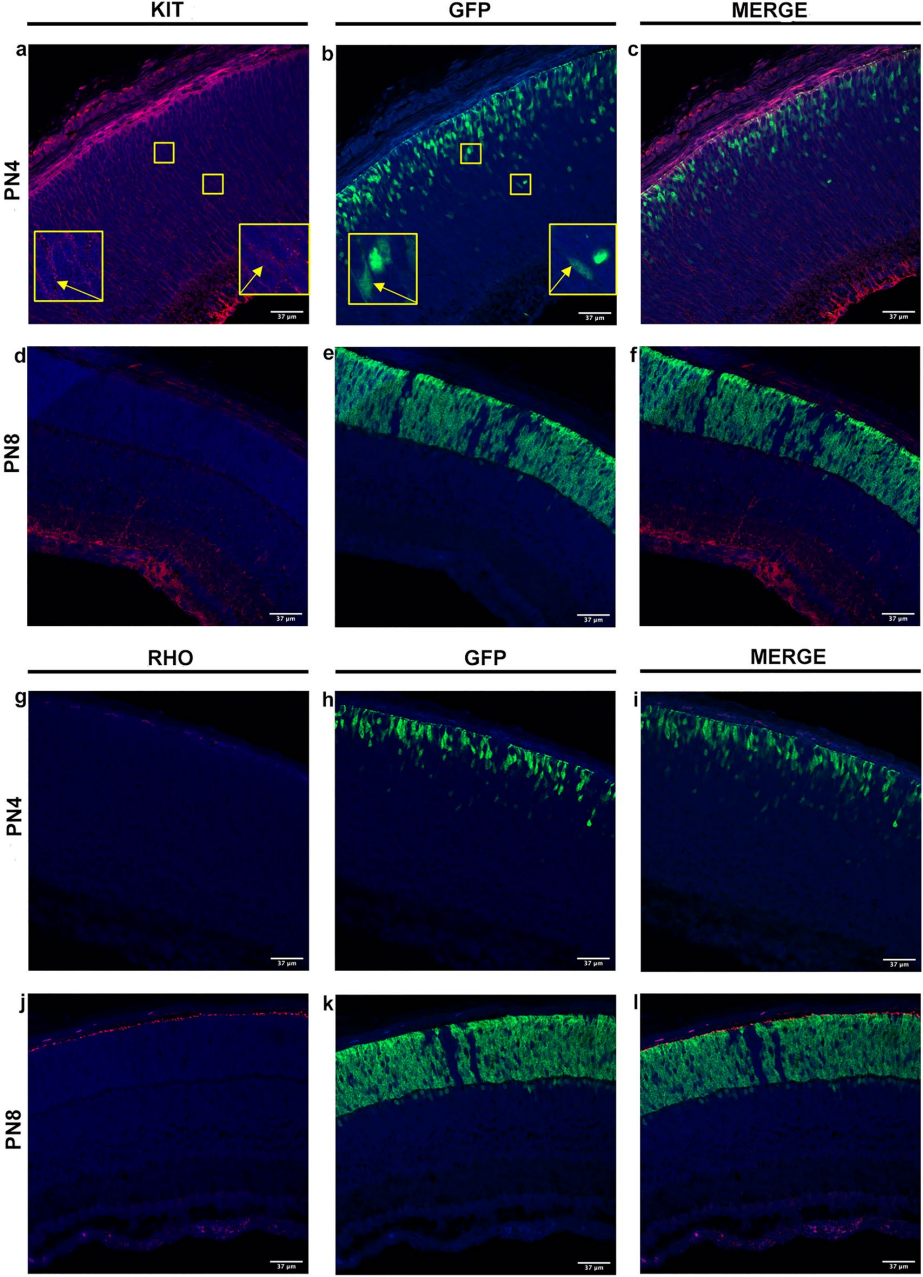
Confocal immunofluorescence imaging of c-Kit and Rho in PN4 and PN8 NRL-GFP^+^ retina. (**a**–**f**) Immunostaining for c-Kit at PN4 (**a**) and PN8 (**d**). GFP expression at PN4 (**b**) and PN8 (**e**). Merged images at PN4 (**c**) and PN8 (**f**). Insets in panels (**a**) and (**b**) show a magnification of GFP^+^ cell labelling positive for c-Kit. (**g**–**l**) Rho immunostaining at PN4 (**g**) and PN8 (**j**). GFP signal at PN4 (**h**) and PN8 (**k**). Merged images at PN4 (**i**) and PN8 (**l**). The calibration bar corresponds to 37 µm.

Using immunostaining, we assessed whether transcriptomic data indicating changes in *c-Kit* and *Rho* expression translated into changes in protein expression. In PN4 retinal slices, c-Kit immunostaining confirmed the labelling of cells in the neuroblast layer (Fig. 2a), including GFP^+^ rod precursors residing in its outermost part (Fig. 2c). Furthermore, in PN8 GFP^+^ cells, the lack of a c-Kit label in the ONL (Fig. 2d,e) matched *c-Kit* down-regulation by cells that had already started expressing Rho (Fig. 2j–l), consistent with *Rho* upregulation between PN4 and PN8 (Supplementary Table S1).

### An increase in cell culture density selectively downregulates the expression by rod precursors of MG genes

Diffusible signals generated by retinal neurons may operate as extrinsic factors controlling rod precursors fate stabilisation^9–11^ as a function of cell culture density.

A recent analysis of retinal stiffness indicates a gradient across layers, increasing with the number of cells for unit volume from the ganglion cells layer to the ONL in both ruminants^37^ and mouse retinas^38^. Accordingly, ONL stiffness is expected to increase during development, initially due to the increase in rod cell number, then in response to junctional complex formation between rods and MGCs, and finally by the adhesion of outer segments to the stiffer RPE-choroid complex.

**Figure 3:**
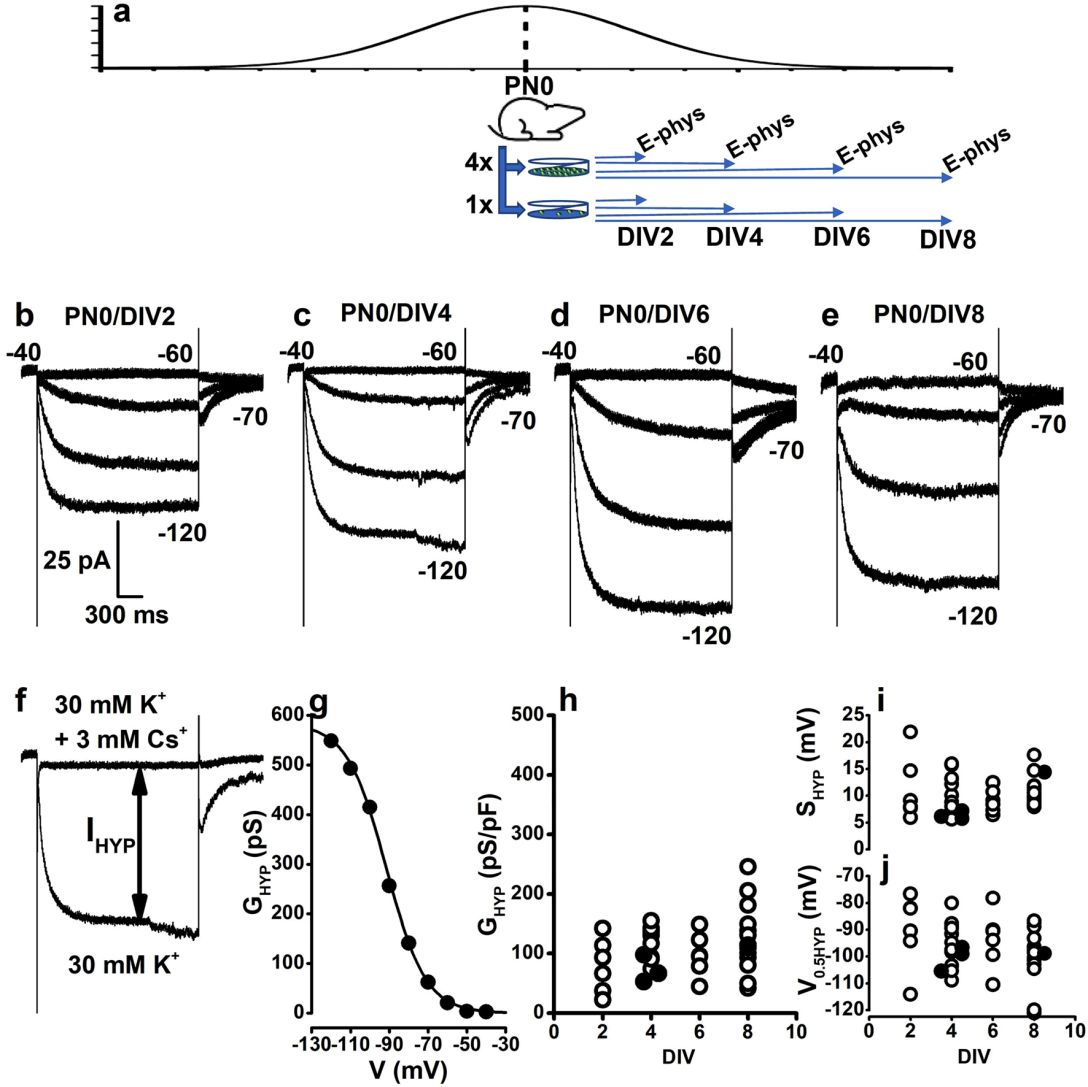
Functional properties of cultured rod precursors. (**a**) The Y-axis plots the rod precursor generation rate in arbitrary units. The scheme illustrates the generation of retinal cultures at 1 × and 4 × cell densities from PN0 mice. Horizontal arrows indicate the use of DIV2, DIV4, DIV6, and DIV8 cultures for patch-clamp recordings (E-phys). (**b**–**d**) Sweeps plot membrane currents activated in response to 2 s-long voltage steps at − 60, − 80, − 100, and − 120 mV from a holding voltage of − 40 mV in GFP^+^ cells isolated on the day of birth (PN0) and recorded after 2 (PN0/DIV2) (**b**), 4 (PN0/DIV4) (**c**), 6 (PN0/DIV6) (**d**) and 8 (PN0/DIV8) (**e**) days in vitro (DIV). (**f**) Protocol for measuring the Cs-sensitive current (I_HYP_) at steady-state (see “Materials and methods”). The double arrows line indicates the current activated by membrane hyperpolarisation (I_HYP_), computed by the difference between current amplitudes in saline with either 30 mM KCl or 30 mM KCl + 3 mM CsCl. (**g**) Filled symbols plot membrane conductance (G_HYP_) values generated by diving I_HYP_ for the driving force (see “Materials and methods”). G_HYP_ is plotted, after normalisation to membrane capacitance, as a function of activating voltage. The continuous line plots the best-fitting Boltzmann function used to estimate activation parameters (see “Materials and methods”) for the PN0/DIV4 cell above. (**h**–**j**) Symbols plot parameters generated by best-fits to individual cells voltage-dependent activation curves similar to panel (**f**). Circles plot G_HYP_ (**h**) normalised to membrane capacitance, the inverse slope factor S_HYP_ (**i**), and the half-activation voltage V_0.5HYP_ (**j**) for PN0/DIV2 (N = 6), PNO/DIV4 (N = 11), PN0/DIV6 (N = 6), and PN0/DIV8 (N = 13) cells cultured at 1 × (open circles); PN0/DIV4 (N = 3) and PN0/DIV8 (N = 1) cells cultured at 4 × cell density (filled circles). Two-way ANOVA indicated non-significant effects of DIV (F = 1.42953 with 3, 32 df: P = 0.25233) and cell density (F = 1.11282 with 1 and 32 df: P = 0.29937) on G_HYP_.

To assess the role played on rod vs MG fate stabilization by factors linked to the number of cells for surface area/volume, we disrupted cell–cell contacts by enzymatic treatment followed by mechanical dissociation and plated isolated retinal cells at either low (1 ×—9.4 × 10^4^ cells/cm^2^) or high (4×—3.77 × 10^5^ cells/cm^2^) cell densities (see also “Materials and methods”). As shown in Fig. 3a, we selected the day of birth (PN0) for cell isolation to ensure enough cells for subsequent analysis and a timespan to adapt to the in vitro conditions and develop a response to cell culture density. As a preliminary step, we evaluated whether isolated rod precursors maintain their electrophysiological identity in culture by monitoring the Cs-sensitive hyperpolarisation-activated current (I_HYP_), a prominent inward current of adult rods expressed by both mice^39^ and human rod precursors^40^. A substantial fraction of I_HYP_ flows through ion channels coded by *Hcn1*, a gene already expressed at an early developmental time and whose expression progressively builds up till PN28 (adult). Although *Hcn1* is not a rod-specific gene and is not among the genes most upregulated between PN4 and PN8, it is expressed by most primary sensory neurons^26^, and I_HYP_ measurement may indicate whether rod precursors maintain their sensory neuron phenotype in culture. Furthermore, recent evidence in human rod precursors of iPSC-derived retinal organoids indicates that the current carried by HCN1 channels is sensitive to 4-hydroxy tamoxifen^40^, a blocker of estrogen-related receptor beta (ERRβ) critical for rod viability^41^ coded by the DEG *Esrrb*.

As shown in Fig. 3b–e, patch-clamp recordings indicated the broadly similar electrophysiological identity of GFP^+^ rod precursors isolated from PN0 mice and cultured at 1 × cell culture density for 2 (Fig. 3b), 4 (Fig. 3c), 6 (Fig. 3d), and 8 (Fig. 3e) days in vitro (DIV), henceforth referred to as PN0/DIV2, PN0/DIV4, PN0/DIV6 and PN0/DIV8, respectively. Analysis of the Cs-sensitive membrane conductance G_HYP_ (Fig. 3h, see figure legend) indicated a modest increase in PN0/DIV8, consistent with the slow progressive increase in *Hcn1* expression shown by transcriptomic data^24^. In addition, developmental time in vitro did not significantly impact the half-activation voltage (V_0.5_) (Fig. 3i). Similar activation parameters were found in GFP^+^ rod precursors recorded in 4 × cell culture density (filled circles in Fig. 3h–j).

Surprisingly, 5 out of 19 PN0/DIV8 GFP^+^ cells lacked I_HYP_ and had an electrophysiological signature akin to MG (Supplementary Fig. S1). This unexpected observation may indicate that some rod-fated precursors in 1 × cell density cultures deviate from their initial fate assignment, drifting toward the MG fate, a notion consistent with the hybrid transcriptional profile of sorted GFP^+^ rod precursors, which express *c-Kit* and *Mcam* along rod-specific genes up to PN4.

A critical issue with transcriptomic data from pooled rod precursors generated across a broad developmental window^4,42^ is that they may provide a blurred picture of the intrinsic dynamics of single rod precursors. For instance, there is no evidence that a specific *c-Kit*-expressing cell in a pooled cell population would also express the rod-specific genes *Rho* or *Mcam*, a gene critical for MG development^36^. Furthermore, rod precursors expressing GFP in response to Nrl may eventually deviate from their initial fate assignment and start expressing genes of different fates while maintaining the GFP fluorescence due to the high protein stability.

**Figure 4:**
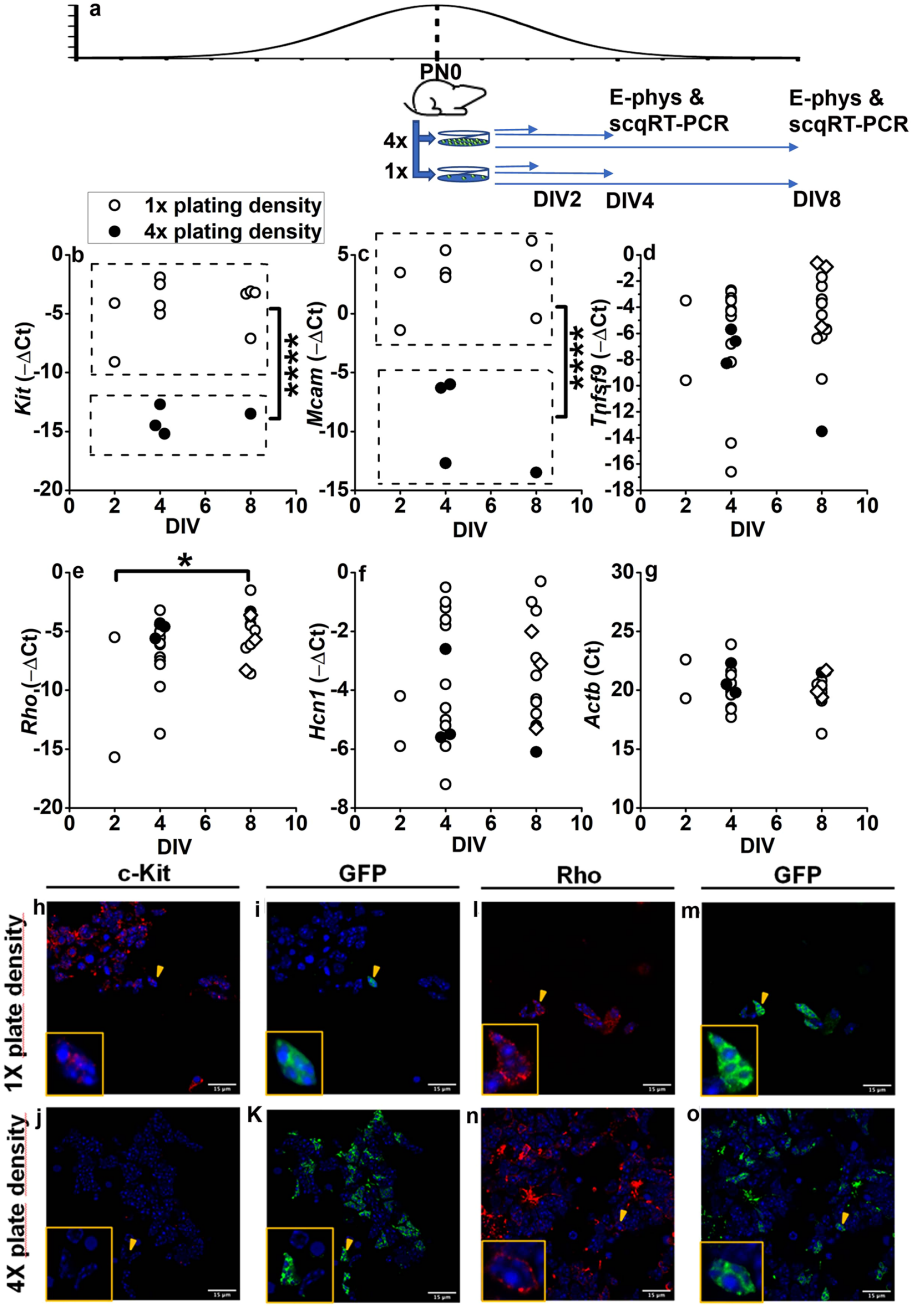
Single-cell qRT-PCR and immune-cytochemistry analysis of cell culture density impact in rod precursors. The scheme illustrates the generation of 1 × and 4 × cell culture densities from PN0 mice. Horizontal arrows indicate the time of patch-clamp recordings (E-phys) and cell collection for single-cell quantitative RT-PCR (E-phys and scqRT-PCR) from DIV2, DIV4, and DIV8 cultures; (**b**–**e**) circles plot − ΔC_t_ values—(C_tGene_ − C_tActb_); (see “Materials and methods”—“Single-cell real-time qRT-PCR”) for c-Kit (N = 14) (**b**), Mcam (N = 12) (**c**), Tnfsf9 (N = 29) (**d**), Rho (N = 29) (**e**) and Hcn1 (N = 29) (**f**) in 29 functionally-characterized single GFP^+^ rod precursors. (**g**) C_t_ values for the housekeeping gene Actb (N = 29). Open and filled circles plot data from cells cultured at lower (1 ×) and higher (4 ×) densities, respectively (see “Materials and methods”). Diamonds plot − ΔC_t_ values for GFP^+^ cells with functional properties akin to MG (see text and Supplementary Fig. S1). *****P < 0.00001 and ***P < 0.001 for c-Kit and Mcam, respectively. Two-way ANOVA for the effects of DIV and cell density (for cell density, F = 70.55 with 1, 10 df for c-Kit and F = 45.02 with 1, 8 df for Mcam) followed by Bonferroni’s test). *P < 0.05 for the effect of DIV on Rho expression by two-way ANOVA for the effects of DIV and cell density (F = 3.96 with 2 and 22 df), followed by Bonferroni’s test for multiple comparisons indicating a significant difference (t = − 2.74594 with 2, 14 df: P = 0.03538) for ΔC_t_ average difference between PN0/DIV2 and PN0/DIV8 cells (− 5.87 ± 2.15). (**h**–**k**) c-Kit immunostaining of DIV8 cells plated at 1 × (**h**) and 4 × (**j**) cell culture density along with GFP signal (**i**,**k**). Arrowheads point to cells shown at an expanded scale in the inset. Note the lack of c-Kit staining in cells plated at 4 × density compared to the 1 × cells. (**l**–**o**) Rho staining of DIV8 cells plated at 1 × (**l**) and 4 × (**n**) density along with GFP signal (**m**,**o**). Arrowheads point to cells shown at an expanded scale in the inset. Note Rho similar staining in cells plated at 4 × and 1 × cell density.

To circumvent these possible issues, we measured the expression of down-regulated genes such as *c-Kit*, *Mcam*, and *Tnfsf9* along with the rod-specific gene *Rho* and *Hcn1* in single GFP^+^ cells functionally characterised by patch-clamp recording and cultured at either 1 × or 4 × cell densities. As shown in Fig. 4a, assuming a similar developmental time in vivo and in vitro, we collected PN0/DIV2, PN0/DIV4 and PN0/DIV8 GFP^+^ rod precursors for single-cell qRT-PCR. As shown by data in Fig. 4, both *c-Kit* (Fig. 4b) and *Mcam* (Fig. 4c) expression remained high in PN0/8DIV GFP^+^ cells cultured at the 1 × cell culture density (open circles), but their expression decreased significantly (***P < 0.001, see figure legend) in cells cultured at the 4 × cell density (filled circles).

The influence of cell culture density appears specific, with no effect on − ΔC_t_ of *Tnfsf9* (Fig. 4d), another gene down-regulated during rod specification. Cell density affected neither *Rho* (Fig. 4e) nor the housekeeping gene *Actb* (Fig. 4g) expression, but *Rho* expression significantly increased with time in culture (*P < 0.05, for the comparison between PN0/DIV2 and PN0/DIV8, see legend) independently of cell culture density.

Consistent with single-cell expression data, immunostaining showed c-Kit labelling of several PN0/DIV8 cells cultured at the 1 × cell culture density, including the GFP^+^ cell (yellow arrowheads in Fig. 4h,i), plotted at higher magnification in the inset. On the other hand, PN0/DIV8 cells cultured at the 4 × cell culture density did not exhibit c-Kit labelling, either by GFP^+^ or GFP^−^ cells (Fig. 4j). In agreement with single-cell data, cell culture density does not affect Rho labelling in PN0/DIV8 cells.

**Figure 5:**
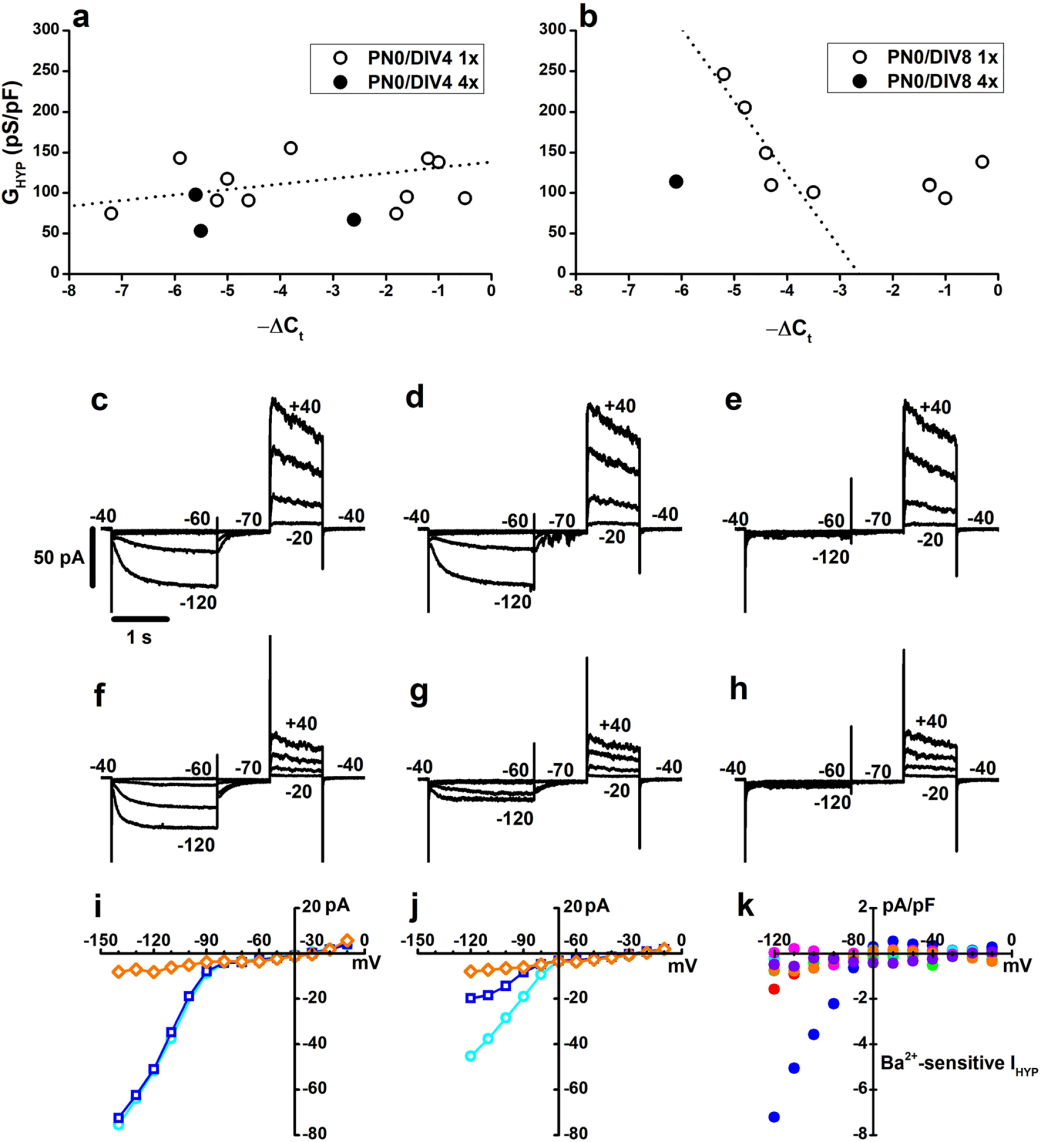
Correlation between gene expression and membrane conductance in single rod precursors. (**a**,**b**) Circles plot G_HYP_ as a function of Hcn1 *ΔC*_*t*_ for PN0/DIV4 (**a**) and PN0/DIV8 (**b**) GFP^+^ cells. Dotted lines plot best-fitting straight lines to GFP^+^ cultured at the lower density (open circles). (**c**–**h**) Sweeps plot currents activated by hyperpolarising and depolarising voltage steps in PN0/DIV8 GFP^+^ cells, as indicated by numbers close to the sweeps, in saline with 30 mM KCl (**c**,**f**), in saline with 30 mM KCl + 2 mM BaCl_2_ (**d**,**g**), and saline with 30 mM KCl, 2 mM BaCl_2_ and 3 mM CsCl to block the residual I_h_. (**i**,**j**) I/V curves plot membrane currents measured at the end of the 2 s-long hyperpolarization steps in 30 mM KCl (cyan circles), 30 mM KCl + 2 mM BaCl_2_ (blue squares), 30 mM KCl + 2 mM BaCl_2_ + 3 mM CsCl (orange diamonds) for the cell in (**c**–**e**) (**i**) and in (**f**–**h**) (**j**). (**k**) Circles plot the amplitude of the BaCl_2_-sensitive current in 6 PN0/DIV8 GFP cells cultured at the 1 × cell density. Current amplitudes have been normalised by cell membrane capacitance.

Consistent with data in Fig. 3h indicating a non-significant increase in G_HYP_ in PN0/DIV8 cells, *Hcn1* expression did not significantly increase with time in culture (Fig. 4f). However, the observation that PN0/DIV8 GFP^+^ cells with an electrophysiological profile of MG-like cells, i.e., lacking I_HYP_ (Supplementary Fig. S1), had − ΔC_t_ values for *Hcn1* (open diamonds) similar to rod-like cells (circles) (Fig. 4e), suggests a mismatch between *Hcn1* expression and normalised Cs-sensitive G_HYP_. We investigated this mismatch by plotting in Fig. 5 normalised G_HYP_ as a function of − ΔC_t_ values separately for PN0/DIV4 and PN0/DIV8 rod precursors cultured at 1 × cell density. G_HYP_ values of PN0/DIV4 cells cultured at the 1 × density (Fig. 5a, open circles) showed a moderate increase with *Hcn1* − ΔC_t_ values spanning from − 7.2 to − 0.5, i.e. an over 2-log_10_ units change in *Hcn1* expression.

Intriguingly, normalised Cs-sensitive G_HYP_ values showed a significant decrease with *Hcn1* − ΔC_t_ values in PN0/DIV8 rod precursors cultured at the 1 × cell density (Fig. 5b).

We evaluated the presence of a second Cs-sensitive inward rectifying current (I_ir_) as a possible cause of the inverse relationship between G_HYP_ and *Hcn1* expression in a fraction of GFP^+^ cells. This notion is intriguing because *Kcnj10*, which codes for inward rectifying potassium channels (K_ir_4.1), appears not dispensable for the MG cell fate, and its expression progressively increases during MG postnatal development^36^.

Figure 5 shows the impact of Ba^2+^, a K_ir_ channels blocker that does not affect HCN1 channels (I_h_), on I_HYP_ of PN0/DIV8 cells cultured at the 1 × cell density (Fig. 5c–h). Ba^2+^ action ranges from a lack of effect (Fig. 5c–e) to an over 50% block of I_HYP_ (Fig. 5f,g), with the residual I_h_ abolished by Cs (Fig. 5h).

In 6 rod precursors, Ba^2+^ blocked a variable fraction of I_HYP_ (Fig. 5k), suggesting that PN0/DIV8 cells cultured at 1 × cell density (Fig. 5b) represent rod precursors caught while smoothly transitioning toward a hybrid cell type displaying electrophysiological fingerprints intermediate between rod precursors and MG. In line with the notion of *c-Kit* and *Mcam* suppression in response to an increase in cell culture density, the only PN0/DIV8 GFP^+^ cell cultured at the 4 × density (Fig. 5b, filled circle) had a lower G_HYP_ than cells with similar *Hcn1* − ΔC_t_ but cultured at a lower cell density.

### Cell culture density affects rod precursors maturation over a limited time window

**Figure 6:**
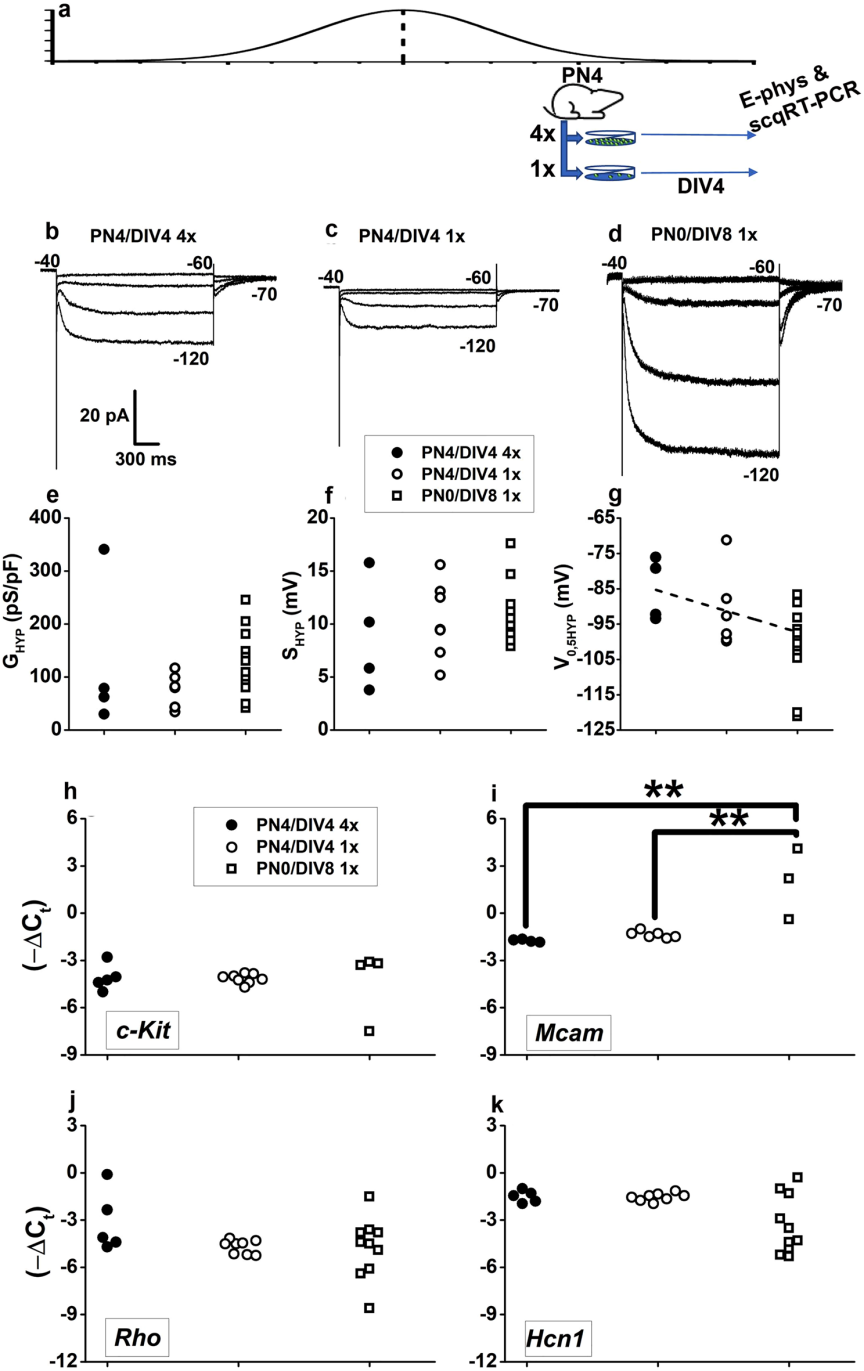
Cell culture density affects rod precursors over a restricted time window. (**a**) The scheme illustrates the generation of 1 × and 4 × cell culture densities from PN4 mice. Horizontal arrows indicate patch-clamp recordings and cell collection for single-cell quantitative RT-PCR (E-phys and scqRT-PCR) in DIV4 cultures; (**b**,**c**) Sweeps plot *I*_*HYP*_ recorded from GFP^+^ cells isolated at PN4 and cultured up to DIV4 (PN4/DIV4) at 4 × (**b**) and 1 × (**c**) cell densities. (**d**) Currents recorded from a GFP^+^ cell isolated at PN0 and cultured up to DIV8 (PN0/DIV8) at the 1 × cell density. (**e**–**g**) Symbols plot Boltzmann’s fits estimates of G_*HYP*_ (maximum conductance) (**e**), S_HYP_ (slope factor) (**f**), and V_0.5HYP_ (half-activation voltage) (**g**) for PN4/DIV4 cells plated at 4 × (filled circles, N = 4) and 1 × cell density (open circles, N = 8). Inset in (**f**) also applies to (**e**) and (**g**). Squares plot activation parameters for PN0/DIV8 cells (N = 13) cultured at 1 × cell density. The dashed line in (**g**) plots the best fit to data points. One-way ANOVA indicates a borderline impact of culturing conditions on V_0.5HYP_ (F = 3.438 with 2 and 20 df; P = 0.052). (**h**–**k**) Circles plot − ΔC_t_ values for c-Kit (**h**), Mcam (**i**), Rho (**j**), and Hcn1 (**k**) in single PN4/DIV4 GFP^+^ cells cultured in 4 × (filled circles, N = 5) or 1 × (open circles, N = 8) cell densities. Open squares plot − ΔC_t_ values for PN0/DIV8 GFP^+^ cells cultured at 1 × density. Inset in (**h**) also applies to (**i**), (**j**) and (**k**).**P < 0.01 by one-way ANOVA for Mcam − ΔCt (F = 13.57 with 2, 10 df: P = 0.001415), followed by multiple comparisons with Bonferroni’s test for 4 × PN4/DIV4 (N = 4) vs 1 × PN0/DIV8 (N = 4) (t = 4.757, P = 0.00224); 1 × PN4/DIV4 (N = 6) vs 1 × PN0/DIV8 (N = 4) (t = 4.608, P = 0.00291); 4 × PN4/DIV4 (N = 4) vs 1 × PN4/DIV4 (N = 4) (t = 0.581, P = 1)*.*

Some PN0/DIV8 GFP^+^ cells cultured at 1 × cell density may escape fate derangement toward a hybrid phenotype, as suggested by their low G_HYP_ with high *Hcn1* expression (Fig. 5b) or by the lack of Ba^2+^-sensitive I_HYP_ (Fig. 5c–e). GFP^+^ cells escaping diversion to the hybrid phenotype may result from cell culture density impacting rod precursors during a restricted time window. Considering that increased cell culture density strongly suppressed *c-Kit* and *Mcam* expression in PN0/DIV4 rod precursors, we investigated whether cell density would impact rod precursors cultured 4-day in vitro following isolation at PN4 (PN4/DIV4), i.e., after they had proceeded up to PN4 through the specification steps in their native retinal environment rather than in culture (Fig. 6a), but before the occurrence of the transcriptional switch at PN6^24^.

Figure 6 shows I_HYP_ recorded from two PN4/DIV4 GFP^+^ cells cultured at 4 × (Fig. 6b) and 1 × (Fig. 6c) cell densities and, for comparison, the PN0/DIV8 GFP^+^ cell from Fig. 3 cultured at the 1 × cell density (Fig. 6d). Analysis of activation parameters indicated a trend toward G_HYP_ reduction in PN4/DIV4 cells (Fig. 6e). In addition, the half-activation voltage (V_0.5HYP_) of PN4/DIV4 cells cultured at the 4 × cell density (filled circles) (Fig. 6g) shows a borderline shift (see legend) toward less negative V_0.5_. According to data in Fig. 5, the occurrence of GFP^+^ precursors downregulating *Hcn1* expression while shifting toward MG-like cells by upregulating current through Ba^2+^-sensitive Kir channels may increase G_HYP_ variability in PN0/DIV8 compared to PN4/DIV4 cells. The observation of similar variability in the normalized conductance of PN4/DIV4 cells at the two culture densities suggests that most rod precursors at PN4 have lost their sensitivity to cell culture density.

This notion is consistent with gene expression analysis comparing PN4/DIV4 to PN0/DIV8 cells (Fig. 6h–k). At variance with data collected in PN0/DIV4 rod precursors in Fig. 4, c-*Kit* (Fig. 6h) and *Mcam* (Fig. 6i) expression in PN4/DIV4 cells did not change with culture cell densities. However, PN4/DIV4 cells (circles) show a significantly reduced (*P < 0.05, see legend) *Mcam* expression compared to PN0/DIV8 cells cultured at 1 × cell density (squares, Fig. 6i). On the other hand, cells isolated at PN4/DIV4 cells (circles) had − ΔC_t_ values for *c-Kit* (Fig. 6h) not significantly different from those of PN0/DIV8 cells (squares) cultured at 1 × cell density. It is relevant to note that even for *Mcam,* the reduction in its expression in PN4/DIV4 cells is far less pronounced than that observed in PN0/DIV4 cells for changes in cell density. Overall, these observations suggest a substantially decreased sensitivity to changes in culture cell density by cells isolated at PN4 compared to those isolated at PN0.

In PN4/DIV4 cells (circles), both *Rho* (Fig. 6j) and *Hcn1* (Fig. 6k) − ΔC_t_ values were similar at either culture densities and not significantly different from those of PN0/DIV8 GFP^+^ cells cultured at low cell density (squares).

**Figure 7:**
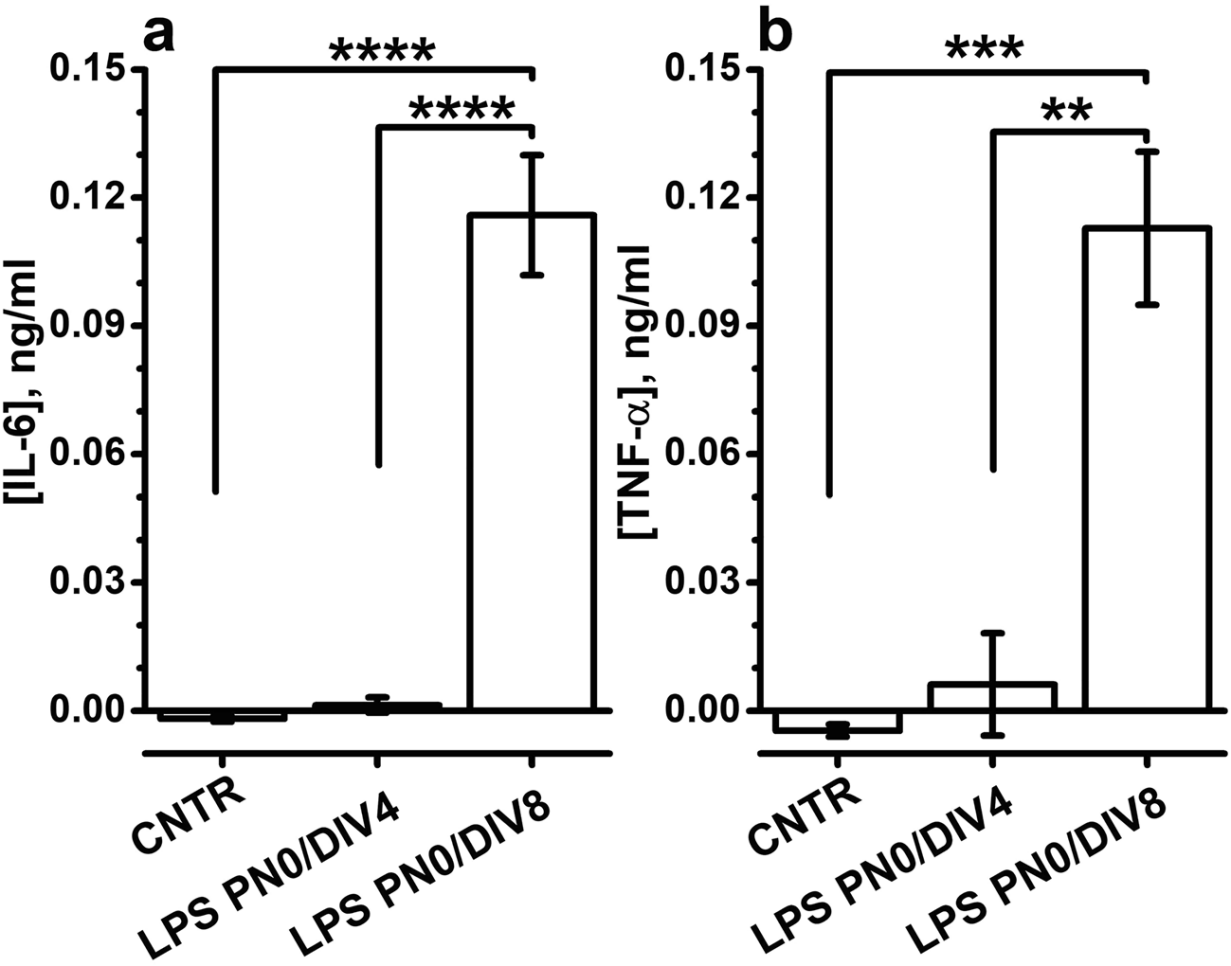
Low cell culture density provides permissive conditions for MG properties. (**a**,**b**) Columns plot IL-6 (**a**) and TNF-α (**b**) concentrations in culture medium collected at DIV4 and DIV8 after 24 incubation with 10 µg/ml LPS. CNTR plot pooled values from untreated wells at DIV4 and DIV8. ****P < 0.0001 and ***P < 0.001, respectively, by one-way ANOVA (F = 201.37909 with 2, 6 df: P = 3.16267 × 10^–6^) for (**a**) and (F = 39.70053 with 2, 6 df: P = 3.46788 × 10^–4^) for (**b**). Multiple comparisons with Bonferroni’s test*: *(**a**) CNTR vs LPS PN0/DIV4 t = 0.4806, P = 1; CNTR vs LPS PN0/DIV8 t = 17.61544, P = 6.44505 × 10^–6^; LPS PN0/DIV4 vs LPS PNO/DIV8 t = 17.13484, P = 6.29031 × 10^–6^. (**b**) CNTR vs LPS PN0/DIV4 t = 1.62042, P = 0.46881; CNTR vs LPS PN0/DIV8 t = 8.39846, P = 4.65651 × 10^–4^; LPS PN0/DIV4 vs LPS PNO/DIV8 t = 6.77804, P = 0.00151.

Data in Fig. 5, showing the increase in Ba^2+^-sensitive G_HYP_ in PN0/DIV8 cells in the 1 × cell culture density, suggest that the low cell culture density may not simply fail to switch off the expression of genes critical for MG cells development, but it may promote the development of MG functional properties. To provide additional evidence for this point, we exploited the response of MG to inflammatory stimuli, such as bacterial lipopolysaccharide (LPS), which induced the release of inflammatory cytokines in the medium by both human and mouse MG^43,44^. As shown in Fig. 7, LPS treatment induced a significant increase of inflammatory cytokines IL-6 (Fig. 7a) and TNF-α (Fig. 7b) in the culture medium of PN0/DIV8 compared to untreated controls. Intriguingly, LPS treatment did not trigger cytokines release in the medium by PN0/DIV4 cells, consistent with the notion that 1 × density culture provides permissive conditions for developing MG properties after DIV4.

## Discussion

The notions of fate reassignment in postmitotic cells and extrinsic factors' roles in promoting fate diversion represent unsettled issues.

Transcriptional data in Fig. 1 and Table S1 from pooled rod precursors indicated that GFP^+^ rod precursors (sorted from retinas of Nrl-GFP^+^ transgenic mice) express genes critical for MG development (*Mcam*) and also genes common to retinal progenitors and MG (*c-Kit*) up to PN4. Furthermore, molecular analysis by qRT-PCR in single retinal cells (Fig. 4) indicated the simultaneous expression by GFP^+^ PN0/DIV4 of a retinal progenitor gene (*c-Kit*), as well as of a gene critical for MG cells development (*Mcam*) along with rod-specific (*Rho*) and primary sensory neurons-expressed genes (*Hcn1*). Overall, these findings support the notion of intrinsically hybrid rod precursors and oppose the possibility that transcriptome data in Fig. 1 and Supplementary Table S1 may result from the pooling of GFP^+^ cells at different stages of maturation rather than their intrinsic hybrid features. Furthermore, immunostaining for c-Kit in ONL GFP^+^ cells at PN4 (Fig. 2) and in cultured PN0/DIV4 cells (Fig. 4) suggest ongoing *c-Kit* transcription by GFP^+^ rod precursors, i.e., by bonafide rod-fated cells.

The hybrid transcriptional profile of rod precursors up to PN4, i.e., well after the peak of rod generation at PN0, suggests fate stabilisation does not occur stepwise, providing a window for rod precursors fate reassignment during their early postmitotic development.

We explored cell culture density's role in either fate stabilisation or reassignment by assessing its ability to suppress genes common to MG and late progenitor cell fates and observed a highly-significant suppression by PN0/DIV4 rod precursors of both *c-Kit* and *Mcam* in response to a fourfold increase in cell culture density (Fig. 4). Moreover, cell culture density affected neither the rod-specific gene *Rho* nor *Actb*, suggesting a specific action on downregulated genes of different fates.

The observation that an increase in cell culture density does not suppress the expression of *Tnfsf9*, a gene downregulated during photoreceptor development, may indicate that multiple extrinsic factors control rod precursors' postnatal maturation. Indeed, in the developing mouse retina, Hypoxia-inducible factor-alpha (coded by *Hif1a*) stabilisation by the propyl isomerase inhibitor Roxadustat^45^ upregulates *Tnfsf9* expression in rod precursors, suggesting that O_2_ partial pressure (pO_2_) represents an additional extrinsic factor that shapes the transcriptional profile of rod precursors in response to the development of the choroidal vasculature.

Cell culture density has previously been reported as an extrinsic factor able to redirect rod precursors toward a different late-born neuronal fate^9^. In agreement with the notion of cell density operating within a restricted time window, we found that changes in culture cell density did not significantly affect *c-Kit* and *Mcam* expression by retinal cells isolated at PN4 and cultured 4 days in-vitro (PN4/DIV4), suggesting that rod precursors sensitivity to cell culture density subsides after PN4. In PN4/DIV4 rod precursors, *Mcam* levels fell between those of PN0/DIV4 cells cultured at two different cell densities. This observation may indicate rod precursors maturing in the native retinal environment had already started suppressing *Mcam *expression by PN4, but without reaching the extent observed in the 2 × culture conditions, in agreement with data in Supplementary Table S1 and Fig. 2 showing *c-Kit* expression and immunostaining in the retina up to PN4. The observation of similar *c-Kit* expression levels in PN4/DIV4 and PN0/DIV8 rod precursors cultured at the 1 × cell density may indicate a different sensitivity to cell culture density between *Mcam* and *c-Kit*.

The effects of cell culture density on rod precursors maturation have been attributed to low molecular weight diffusible factors^9,46^. Taurine^47^ and retinoic acid^48^ have been reported to promote rod precursors maturation or steer retinal progenitors toward the rod fate at the expense of other late retinal-born neurons (reviewed by^7^). However, the impact of cell density in our cultures does not appear to recapitulate the effects of known small molecular weight diffusible factors on rod precursors^9^. First, cell density effects occur irrespectively of cells being exposed to the same retinoic acid level (1 µM in the culture media). In addition, patch-clamp recordings from GFP^+^ cells in Fig. 3 indicate they display functional properties akin to rod precursors rather than bipolar cells, as previously reported for cells cultured in 1 × density^9,46–48^.

Cell–cell contacts and tissue stiffness are two crucial differences between our experimental conditions and those used by Altshuler and Cepko^9^, which embedded dispersed retinal cells in a three-dimensional collagen matrix to avoid cell–cell contacts and provide a soft matrix for cells^9^. On the other hand, we cultured cells as a two-dimensional system, allowing cells to establish cell–cell contacts. Indeed, recent evidence indicates that a stiffness gradient occurs across retinal layers linked to the number of cells per unit volume^37,38^, suggesting that ONL stiffness may progressively increase during development, mirroring the increase in ONL cell number.

We did not investigate in detail the molecular mechanisms underlying cell culture density impact on rod precursors' maturation. However, the expression of transcription factor Yes-associated protein (*Yap1*), known for its role in mechanotransduction^49,50^, was not detectable in PN4/DIV4 GFP^+^ cells, a finding consistent with the downregulated *Yap1* expression found between PN4 and PN10 in rod precursors transcriptomic data^24^. Yap1 is expressed in adult MG cells^51^, where the Hippo pathway inhibits its nuclear translocation^50,52^ to prevent MG cells proliferation, but it is presently unknown whether Yap signalling in rod precursors may depend on Mcam, as recently reported for glioblastoma cells^53^. Although comparing the transcriptome of sorted PN0/DIV4 GFP^+^ rod precursors cultured using different cell densities may hint at the underlying molecular events, single-cell transcriptome analysis may avoid the shortcomings of substantial cell loss when sorting cultured GFP^+^ rod precursors.

An intriguing point is whether 1 × cell culture density may only prevent rod precursors from switching off the expression of immature genes, such as *Mcam* and *c-Kit*, or it may promote the MG fate. Data in Fig. 7 indicate that low cell culture density provides permissive conditions for developing MG functional properties in PN0/DIV8 retinal cells, although they do not prove that may happen in rod precursors. However, the analysis of P0/DIV8 rod precursors cultured at 1 × cell density revealed that 5 out of 19 GFP^+^ cells developed functional properties akin to Muller glial cells, as judged by the lack of I_HYP_ in response to membrane hyperpolarisation, while *Rho*, *Hcn1,* and *Tnfsf9*, expression levels remained similar to GFP^+^ cells displaying typical rod precursors functional features. Interestingly, the lack of I_HYP_ despite *HCN1* expression has previously been reported for rod precursors derived from adult human MG cells^54^, and mouse ciliary margin-derived Nrl-expressing cells also lack Cs-sensitive I_HYP_ despite *Hcn1* expression^55^. These findings suggest that low cell culture density may not just prevent rod precursors from switching-off MG or immaturity genes but may also promote the expression of the functional profile of MG. Furthermore, data in Fig. 5 reveal that in some PN0/DIV8 rod precursors cultured at the 1 × cell density, two distinct current components contribute to I_HYP_. One current is both Ba^2+^- and Cs^+^-sensitive, thus distinct from the second Ba^2+^-insensitive I_HYP_ component carried through HCN1 channels. This finding is intriguing as adult MG cells express *Kcnj10* (alternative name K_ir_4.1), a member of the *Kcnj* family of potassium-selective ion channels^56^ that codes for Ba^2+^ and Cs^+^-sensitive currents^57,58^ and is critical for MG development^36^.

Although rods upregulate *Kcnj14*^59^ (log2 Fold Change = 3.23) during rod precursors maturation, its low basal level at PN4 and limited inactivation suggest *Kcnj14*-coded channels do not contribute to the inactivating Ba^2+^-sensitive component observed in some PN0/DIV8 rod precursors grown in 1 × cell density culture.

These molecular and functional data suggest that some PN0/DIV8 GFP^+^ cells cultured at the 1 × density may progressively derail from the rod fate toward a hybrid cell type with rod precursor features coexisting with MG cell properties.

The notion of cells with a hybrid cell profile has already been proposed for rod precursors of rd7 mice lacking the TF Nr2e3^17^. Since rod precursors and MG usually do not originate from the same terminally-dividing late progenitor^25^, the finding of rod precursors failing to interconvert with MG cells fits the notion that a fate reassignment may only occur between cell pairs originating from terminally-dividing progenitors. Intriguingly, recent evidence based on single-cell transcriptomic in cells isolated from the adult mouse retina indicates the occurrence of a cell cluster with a hybrid photoreceptor-MG feature and the expansion of this cluster in response to retinal degeneration^60^.

The evidence that cell culture density is an extrinsic factor affecting rod precursors maturation may extend beyond developmental biology, impacting the regenerative approach to treating retinal dystrophies. Transplanting either unsorted retinal cells (4 × 10^5^ cells µl^−1^) or donor mouse NRl-GFP rod precursors (2 × 10^5^ cells/µl^−1^) isolated at PN1 leads to similar integration rate into the host retina^61^. However, photoreceptor replacement via photoreceptor precursors transplantation has been challenged by the occurrence of material transfer between donor and host cells via a cytoplasmic exchange rather than by replacement^62–64^ in an environment-dependent manner^65^. Intriguingly, in a model of terminal photoreceptor degeneration, a significant improvement in the yield of truly integrating donor cells has been reported upon increasing the number of sorted transplanted cone precursors for unit volume^66^ from 2 × 10^5^ cells µl^−1^ to 3.33 × 10^5^ cells µl^−1^. For our cultures on a 13 mm round coverslip, assuming cells may pile up to 5 µm height, culture cell density would range from about 7.5 × 10^5^ cells µl^−1^ (4 ×) to 1.9 × 10^5^ cells µl^−1^ (1 ×). These figures suggest that the improvement in transplantation outcome occurs within the range of cell densities affecting the downregulation of immaturity genes in rod precursors. Evaluation of grafted cells may assess the possible derailment of grafted rod precursors toward a hybrid phenotype upon transfer to the subretinal space, i.e. to a location whose stiffness and pO_2_ levels (recently reviewed in^67^) may differ from the ONL, where rod maturation usually occurs.

## Materials and methods

### Animals

To improve breeding efficiency, heterozygous NRL:GFP^+^ transgenic mice (kindly provided to Prof. Vania Broccoli by Prof. A. Swaroop) were bred with Wild Type CD1 (The Jackson Laboratory, Bar Harbor, ME). Animals husbandry and retina isolation did comply with the guidelines for animal research of the Association for Research in Vision and Ophthalmology, the European Community and Italian laws. The protocol, complying with ARRIVE guidelines, was approved by the Italian Ministry of Health ethical committee (authorization N° 45/2016 of January 16, 2016). The animals were genotyped by ear biopsy using the following PCR primers:

- NrlGFP-geno-Fw: 5′CTGAATACAGGGACGACACCAGC3′.
- NrlGFP-geno-Rv: 5′CGTAGGTCAGGGTGGTCACGAG3′.

### Isolation and dissociation of mouse retinas

Eyes enucleated at PN0 or PN4 after cervical dislocation under isoflurane anaesthesia, and isolated retinas were washed in HBSS with 0.1% Gentamicin and 1% Penicillin/Streptomycin. Digestion in HBSS containing 10 U/ml Papain (Worthington Biochemical Corporation, USA), 0.5 mM EDTA, and 1.5 mM cysteine for 12 min at 37 °C, was followed by mechanical dissociation via passages through a 1000 µl micropipette tip. After centrifugation for 10 min at 180×*g*, cells were resuspended in DMEM:F12 medium with 2 mM of l-Glutamine, 1% Penicillin/Streptomycin, 1% N2 supplement, 2% B27 supplement and 0.5 μM Retinoic Acid. Cells were counted by the Trypan blue exclusion method with a Burker Chamber and seeded on 13 mm glass coverslips previously coated with Poly-d-Lysine and 1% Matrigel at two different cell densities: 0.5 × 10^6^ and 2 × 10^6^ cells were spread over the 1.33 cm^2^ glass coverslip, resulting in cell culture densities of 3.77 × 10^5^ cells/cm^2^ (1 × density) and 1.51 × 10^6^ cells/cm^2^ (4 × density) for coverslip, respectively. Cells were routinely cultured for 2–4–6–8 days in vitro (DIV) at 37 °C in a humidified atmosphere of 5% CO_2_, and the medium changed every other day.

### Transcriptome analysis of NRL-GFP cells

Using the GFP signal, NRL-GFP cells from PN4 and PN8 transgenic mice were sorted by a Fluorescence-Activated Cell Sorting (FACS) system. For every time point, the quality and concentration of total RNA extracted from three sorted biological replicas using the RNeasy mini kit (Qiagen) were assessed with NanoDrop (Thermo Scientific). RNA-seq libraries preparation and sequencing were outsourced to IGATech (Udine, Italy). Libraries were generated according to the TruSeq Stranded Total RNA Ribo-Zero Gold protocol (Illumina, San Diego, CA) and sequenced using the Illumina HiSeq2500 platform with a 125-bp paired-ends design. Three biological replicates were generated for each group of transgenic mice. On average, for each sample, we generated more than 30 million paired-end reads with a quality score (Phred-score) > 30. Raw reads were processed to filter out low-quality reads and adaptors using the program Cutadapt and the wrapper tool TrimGalore v0.6.2 (http://www.bioinformatics.babraham.ac.uk/projects/trim_galore/) with default parameters. Clean reads were used to quantify the annotated *Mus* musculus gene models, assembly version 38.87 (http://www.ensembl.org), with the program Salmon^68^.

Briefly, clean reads were mapped to the reference transcriptome using Salmon quant command with default parameters except for the options --numBootstraps 30. Supplementary Table S4 provides data on the number of reads/sample, number of aligned reads, and concordant pair alignment rate.

Differential expression analysis was performed with the Bioconductor package DESeq2^69^ using the pairwise contrast PN8 vs PN4. Genes were considered significantly differentially expressed (SDE) based on a false discovery rate ≤ 0.1.

Gene Ontology (GO) over-representation analysis with Benjamini–Hochberg FDR correction was conducted in R using the package ClusterProfiler (v 4.0.5)^70^.

### Immunofluorescence on retina slices and Immunocytochemistry

Immunofluorescence on retina slices was performed as previously described^71^. In particular, whole eyes were enucleated at PN4 and PN8, immediately fixed in 4% Paraformaldehyde for 2 h and incubated overnight (O/N) in 15% w/v sucrose solution. The next day, eyes were transferred in cryomolds, submerged with Tissue-Tek^®^ O.C.T. Compound, and sectioned using a cryostat. For immunofluorescence staining, glass slides were reheated 20 min at RT, blocked in 10% BSA in PBS 1 × for 2 h at RT, and incubated with the primary antibody at 4 °C. After incubation with the secondary antibody, the sections were mounted with VECTASHIELD^®^ Antifade Mounting Medium with DAPI.

For immunocytochemistry, PN0/DIV4 or PN0/DIV8 retinal cells were seeded on 13 mm glass coverslips at 1 × or 4 × plate densities (see above), washed with PBS 1 ×, fixed with 4% Paraformaldehyde for 15 min, permeabilised with 0.3% Triton-X100 for 5 min, and blocked with block solution (2% BSA in 1 × PBS–0.1% Tween 20) for 30 min. After incubation for 1 h at 37 °C with the primary antibody at 4 °C, followed by the secondary antibody and DAPI (1:1000), samples were mounted on microscope slides with AquaPolymount (Polysciences, Warrington, PA, USA).

The following primary antibodies were used: c-Kit (D13A2) XP^®^ Rabbit mAb 1:400 (Cell Signaling^®^, #3074); Rhodopsin (D4B9B) Rabbit mAb 1:400 (Cell Signaling^®^, #27182).

Secondary antibodies: Alexa Fluor 568 donkey anti-rabbit IgG, 1:500 (Molecular Probes, Eugene, OR, USA).

All images were acquired on a Nikon Ti-e microscope with an A1 scanning head and 405/488/561/630 laser lines, using a 40 × water immersion objective (Nikon APO NA 1.25) for tissue imaging and 100 × oil immersion objective (Nikon APO TIRF NA 1.49). Zoom and image dimensions were adjusted to obtain a pixel size of 80 nm, and the pinhole was set at Airy 1. A 2 × 2 binning was performed on the acquired images. Laser power was maintained below 0.5% to minimize photobleaching.

### Electrophysiological recordings from cultured rod precursors

A coverslip was transferred to a recording chamber bottomed by a 0.1 mm-thick coverslip mounted on the stage of an inverted microscope equipped with a 63 × objective, a cooled CCD camera, and GFP fluorescence excitation/emission filters. A manifold with electrovalves controlled the switch between saline solutions used to perfuse cells during recording. GFP^+^ rod precursors cells were approached by the patch-pipette using a motorised micromanipulator. Brief treatment with 0.5 mg/ml hyaluronidase increased the success rate of gigaseal formation with cells cultured at 4 × cell density.

We used the perforated-patch technique to prevent I_h_ rundown during whole-cell recording, as previously reported for adult mouse rods^72–74^ and ciliary margin-derived cells^55^. Patch pipettes were drawn from 1.5 mm OD borosilicate glass (Hirschmann) by an air-cooled two-stage horizontal puller, with series resistances ranging from 50 to 80 MΩ, which proved satisfactory for cells with resistances higher than 3 GΩ, membrane capacitance 3–7 pF, and currents with activation time constants longer than 50 ms. When collecting samples for single-cell qRT-PCR, we controlled the filling level of the pipette with the pseudo-intracellular solution to minimise either dilution or concentration of mRNA.

Voltage-gated currents were filtered at 300 Hz by an EPC-8 amplifier, and currents acquired at 1 kHz sampling rate using a 16-bit A/D board were stored in the computer hard disk for later analysis by Origin 8.5.1 Pro (Microcal).

Recordings started in Locke’s solution, an isotonic saline solution allowing both adult and immature mouse rods survival for several hours^55,74^. High K saline used to amplify I_HYP_ contained (in mM): NaCl, 120; KCl, 25; CaCl_2_, 2, MgCl_2_, 2.4; glucose 10; Hepes, 10—pH 7.4. I_HYP_ block was induced by a high K solution with either CsCl or BaCl_2_ to final concentrations of 3 or 2 mM. Patch-pipetted were filled with a pseudo-intracellular solution containing (in mM): NaCl, 10; KCl, 140; Pipes, 10—pH 7.2 with KOH. All salts were from Sigma-Aldrich, Italy.

### Data analysis

For I_HYP_ analysis, we averaged the last 200 ms of 2 s-long steps at voltages ranging from − 10 to − 120 mV and computed net I_HYP_ amplitudes by subtracting average I_HYP_ in high K + CsCl from the value in high K saline at each activating potential. Membrane conductance (*G*_*HYP*_) was computed according to $G_{HYP} \;(V) = \frac{{I_{HYP} }}{{V - E_{HYP} }}$ using a reversal potential (*E*_*HYP*_) of − 30 mV.

To compute the activation parameters: maximum conductance (*G*_*MAX*_), half-activation voltage (*V0.5*), and inverse slope factor (*S*), *G*_*HYP*_*(V)* values were interpolated by a modified Boltzmann equation using a routine in Origin 8.5 (Microcal, OR) according to $G_{HYP} \;(V) = \frac{{G_{MAX} }}{{1 + e^{{ - \frac{{(V - V_{0.5} )}}{S}}} }}$.

G_MAX_ was divided by the cell electrical capacitance (*C*_*m*_), an index of the plasma membrane surface, to account for the difference in cell size. Membrane capacitance (*C*_*m*_) was estimated from total charge (*ΔQ*) computed by numerical integration of current transients evoked by 25 ms-long 20 mV (ΔV) voltage steps from a holding voltage of − 40 mV according to $C_{m} = \frac{\Delta Q}{{\Delta V}}$.

### Single-cell real-time qRT-PCR

At the end of the recording, a single NRL-GFP rod cell was aspirated inside the patch pipette under visual control, carefully avoiding sucking nearby cells. The cell was transferred in a pre-chilled RNase/DNase-free tube filled with 10 ml of Single Cell Lysis/Single Cell DNase I solution (Ambion Single Cell-to-CTTM Kit, Life Technologies, NY) by breaking the pipette tip in the vial cap while applying slight positive pressure. cDNA synthesis and sample pre-amplification were performed in a thermal cycler based on the manufacturer's instructions (Ambion Single Cell-to-CTTM Kit, Life Technologies, NY). Products from the pre-amplification step were diluted 1:10 in Tris–EDTA (TE) buffer and used for the real-time qRT-PCR reaction (50 °C for 2 min, 95 °C for 10 min for enzyme activation, 40 cycles of 95 °C for 5 s and 60 °C for 1 min to denature—anneal/extend), using TaqMan Gene Specific Assays FAM dye-labelled and TaqMan Universal PCR Master Mix. TaqMan Gene Assays are listed in Supplementary Table S5; Actin-beta (*Actb*) was used as the reference gene.

ΔCt values were computed as the difference between the Ct of the gene of interest (Ct_GENE_) and the reference gene (Ct_REF_). Considering the variability of single cell ΔCt values during the early postmitotic development of rod precursors, − ΔCt values are presented as scatter plots rather than average values ± the standard error of the mean (SEM). More negative − ΔCt values correspond to reduced gene expression and, in the case of *Hcn1,* provide a positive correlation with Gh values.

### Analysis of cytokine secretion by dissociated cells after stimulation with LPS

Cells from PN0 retinas were cultured at 1 × cell density and treated at DIV3 or DIV7 for 24 h with 0.1 μg/ml of lipopolysaccharide (LPS). At the end of treatment, 200 μl of culture media were collected, and pro-inflammatory cytokines IL-6 and TNF-α measured by ELISA assay performed according to the manufacturer’s instructions (TNF alpha Mouse ELISA Kit and IL-6 alpha Mouse ELISA KIT, Thermo Fisher Scientific, USA) and expressed as ng/ml. The experiment was performed in biological triplicate.

### Statistical analysis

Except for gene over-representation in GO (see “Transcriptome analysis of NRL-GFP cells” above), data analysis was carried out using Origin 8.5.1. The normality of data distribution was tested by the Shapiro–Wilk test. The impact of time in culture (DIV) and treatment (cell culture density) on I_HYP_ and single-cell expression were analysed by two-way ANOVA. In addition, 1-way ANOVA was used to analyse cytokine secretion in the medium and compare membrane capacitance (Cm) between adult MG cells and PN0/DIV8 cells in Supplementary Fig. S1. Multiple comparisons were carried out using Bonferroni’s correction for a two-tailed *t* test.

### Ethical statement

Animals husbandry and retina isolation did comply with the guidelines for animal research of the Association for Research in Vision and Ophthalmology, the European Community and Italian laws. The protocol, complying with ARRIVE guidelines, was approved by the Italian Ministry of Health (authorization N° 45/2016 of January 16, 2016).

## Supplementary Information

Supplementary Table S1.

Supplementary Table S2.

Supplementary Table S3.

Supplementary Information.

## Data Availability

RNA-seq data were deposited in the NCBI Sequence Read Archive database with the ID PRJNA830838. Data for generating Figures and Tables have been unloaded in the Figshare repository: https://figshare.com/articles/dataset/CELL_CULTURE_DENSITY_HYBRID_ROD-GLIA_CELLS/21333972 (10.6084/m9.figshare.21333972.v3).
