# Supplementary material for: Increasing cell culture density during a developmental window prevents fated rod precursors derailment toward hybrid rod-glia cells
Source: Sci Rep. 2023 Apr 13;13:6025. doi: 10.1038/s41598-023-32571-y (PMC10101963; doi:10.1038/s41598-023-32571-y)
Supplement: Supplementary file 4 — Supplementary Information. [file 41598_2023_32571_MOESM4_ESM.docx]

**INCREASING CELL CULTURE DENSITY DURING A DEVELOPMENTAL WINDOW PREVENTS FATED ROD PRECURSORS DERAILMENT TOWARD HYBRID ROD-GLIA CELLS**

Ivana Barravecchia^1,2^, Chiara De Cesari^2,3^, Viviana Guadagni^3^, Giovanni Signore^3,4^, Edoardo Bertolini^2,5^, Serena Giannelli^6^, Francesca Scebba^2^, Davide Martini^3^, Mario Enrico Pè^2^, Vania Broccoli^6,7^, Massimiliano Andreazzoli^3*^, Debora Angeloni^2*^, Gian Carlo Demontis^1*^.

1) Department of Pharmacy, University of Pisa, Pisa, Italy

2) Scuola Superiore Sant’Anna, Pisa, Italy

3) Department of Biology, University of Pisa, Italy

4) Fondazione Pisana per la Scienza, San Giuliano Terme, Italy

5) Donald Danforth Plant Science Center, St. Louis, USA

6) San Raffaele Hospital, Milan, Italy

7) Institute of Neuroscience, National Research Council of Italy, Milan

*) Corresponding authors

**CORRESPONDENCE:**

**Gian Carlo Demontis, Ph.D.**

University of Pisa,

Department of Pharmacy,

Via Bonanno Pisano, 6

I-56126 Pisa, Italy

Mail: giancarlo.demontis@unipi.it

**Supplementary Table S1 legend.**

In the first column, Gene ID is the Ensemble code. The second, third, and fourth columns report the log2 fold Change (log_2_(PN8/PN4)), its standard error and the base expression in TPM (Transcript Per kilobase Million), respectively. Fifth and sixth columns report the gene name and its symbol, respectively. Eight and ninth columns report transcript p value and gene adjusted value p-adj, respectively.

Genes are listed in Supplementary Table S1 according to decreasing log2FoldChange values.

**Supplementary Table S2: GO terms significantly up- ad down regulated in PN8 vs PN4 rod precursors**

**Supplementary Table S2 legend.** Columns A and B list up- and down-regulated GO terms reported in Figure 1C (A) and their description (B). Column G lists q-values used to generate the heatmap in Figure 1c. Columns I and H list the number of genes upregulated for a given GO term (I) and the Ensemble database ID code for gene upregulated in the specific GO term (H). Column J lists the GO category of a given GO term in the table. Column L indicates whether a given GO term is up- or down-regulated**.**

**Supplementary Table S3. Gene list enrichment analysis in PN8 vs PN4 samples.**

**Supplementary table S3 legend.** Columns A and B list the Ensemble ID code (A) and the gene name (B) for the 12 genes found over-represented with a q-value of 5.89x10^-5^ (K).

**Supplementary Table S4.** Number of reads aligned to the reference *Mus* *musculus* genome

| Sample | Number of reads | Number of reads aligned to the reference genome | Concordant pair alignment rate |
| --- | --- | --- | --- |
| PN8_R1 | 29701865 | 24009488 | 0.808349509 |
| PN8_R2 | 33130662 | 26533873 | 0.800885687 |
| PN8_R3 | 32206162 | 26768570 | 0.831162993 |
| PN4_R1 | 33302530 | 22534748 | 0.676667749 |
| PN4_R2 | 35712202 | 26238532 | 0.734721763 |
| PN4_R3 | 40237255 | 27211655 | 0.676280104 |

**Supplementary Table S4 legend.** Table S4 list, for each sample, the number of reads, the number of reads aligned to the reference genome, and the alignment rate.

**Supplementary Table S5**

| GENE | TaqMan Assay code |
| --- | --- |
| CD137L (or *Tnfsf9)* | Mm 00437155 |
| *Mcam* | Mm 00522397 |
| *Rho* | Mm01184405 |
| *Hcn1* | Mm00468832 |
| *c-Kit* | Mm 00445212 |
| *Yap-1* | Mm01143263 |
| *Actb* | Mm01205647 |

**Supplementary Table S5 legend.** Left column: gene names. Right colum: Taqman Assay code.

**Supplementary Figure S1: Comparison of PN0/DIV8 MG-like cells and MG of the adult mouse retina.**


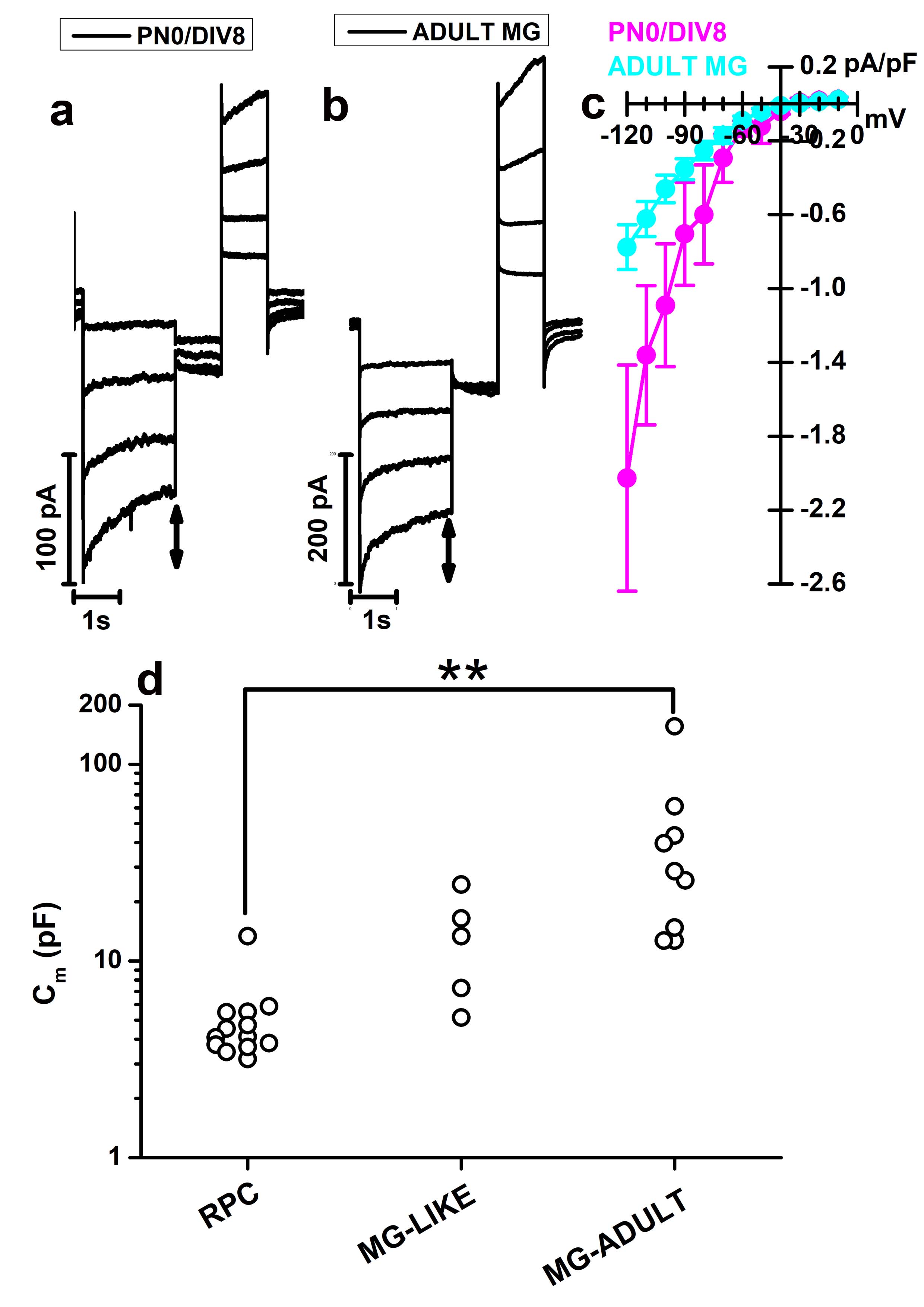


**SUPPLEMENTARY FIGURE S1 LEGEND.**

a) Traces plot the response of a PN0/DIV8 cell, cultured at 1x cell density, to 2s-long hyperpolarising voltage steps at -120, -100, -80, and -60 mV from a holding of -40 mV. The membrane was then stepped to -70 for 500 ms, followed by voltages ranging from -20 to +40 mV. In response to membrane hyperpolarisation, the cell displays fast-activating and slowly-inactivating inward currents (double arrows line). In response to membrane depolarisations positive to -40 mV, the cell generates slowly-activating outward currents. Note the complete lack of both slowly-activating I_HYP_ (see Figure 3 and Figure 5) and slowly-inactivating outward currents (see Figure 5), which provide the electrophysiological fingerprint of both adult rods and rod precursors. b) Traces plot the response to the same voltage protocol in A for an adult MG cell recorded from a 150 µm-thick retinal slice of adult mice. Note the similar time course of fast-activating and slowly-inactivating currents in response to membrane hyperpolarisation in the PN0/DIV8 cell and the adult MG. Also similar are the time course of membrane currents evoked by voltage steps positive to -40 mV. c) Cyan and magenta circles plot the average currents inactivated by membrane hyperpolarisation current in 5 PN0/DIV8 cells (cyan) and 9 MG (magenta) and vertical line plot ±SEM. Currents were measured as the difference in amplitudes at 125 ms (average over 100-150 ms) and 1900 ms (average over 1800-2000 ms) after application of the hyperpolarising voltage steps. Before averaging, data were normalised to the cell membrane capacitance. Note the smaller average normalised currents in (c) for MG than for PN0/DIV8 cells, although current amplitudes were more prominent in the MG cell in (b) than in the PN0/DIV8 cell in (a). The normalised current difference reflects the substantially larger membrane capacitance of MG cells, as show in (d), which plots membrane capacitance (C_m_) for PN0/DIV8 rod precursors (RPC), PN0/DIV8 MG-like cells (MG-LIKE) and adult MG (MG-ADULT). Due to MG-adult having higher C_m_ (43,86±15.02 pF, N=9) than MG-like PN0/DIV8 (13.35±3.45 pF, N=5) cells, MG normalized current is smaller than that of MG-like cells. One-way ANOVA indicates significant differences between groups (F=5.97225 with 2, 24 df: P=0.00785). **, multiple comparisons using Bonferroni’s correction indicate a significant difference between average C_m_ values of PN0/DIV8 RPC (5.04±0.73 pF, N=13) and adult MG (t=3.4068: P=0.00696). PN0/DIV8 MG-like cells average C_m_ values fell between RPC and adult MG, and the differences were not significant (t=0.609: P=1 for PN0/DIV8 RPC vs PN0/DIV8 MG-like; t=2.0816: P=0.14365 for PN0/DIV8 MG-like vs MG adult). Data were low-pass filtered at 300 Hz by a 3-pole Bessel filter and sampled a 1kHz by the A/D board as reported in METHODS.
